# Long-term antidepressant treatment in general practice: changes in body mass index

**DOI:** 10.1192/pb.bp.115.052472

**Published:** 2016-12

**Authors:** Laura Chiwanda, Matthew Cordiner, Anne T. Thompson, Polash Shajahan

**Affiliations:** 1 NHS Lanarkshire, Scotland, UK

## Abstract

**Aims and method** To discern changes in body mass index (BMI) in patients on long-term antidepressant treatment in a general practice population and establish BMI changes in patients with and without a diagnosis of diabetes. We used a retrospective observational method and identified patients on four antidepressants of interest. We excluded those who did not have start and current BMI readings within the past 3 years and noted whether or not patients had a diagnosis of diabetes.

**Results** Long-term treatment with citalopram, fluoxetine, mirtazapine and sertraline was associated with increased BMI in two-thirds of patients. There was reduction in BMI in patients with diabetes and an increase in BMI for patients who did not have diabetes.

**Clinical implications** Awareness of environmental factors and their impact on individuals is important. Medication is not the only cause of abnormal metabolic effects. Overall monitoring of physical health is important in all groups of patients.

Evidence from short-term studies and meta-analyses suggests that there is an association between antidepressant use and weight gain, particularly for mirtazapine.^1,2^ A prescription register study in Finland showed that all antidepressants were associated with weight gain and type 2 diabetes over 1-year follow-up,^3^ although it is unlikely that antidepressants directly cause diabetes mellitus.^4^ When initiating antidepressant therapy, our local guidelines suggest four options – fluoxetine, citalopram, mirtazapine and sertraline.^5^ An important reason for discontinuing antidepressant therapy is side-effects such as weight gain,^6^ however, developing literature in adherence also suggests that side-effects have less importance than originally thought and may not be a primary cause for discontinuation.^7^

Our primary hypothesis was that mirtazapine would be associated with increased weight gain compared with the other antidepressants. We also aimed to establish whether there was an association between long-term antidepressant treatment and changes in weight expressed as changes in body mass index (BMI). As diabetes mellitus is common in patients with depressive disorders,^8^ we also aimed to examine whether patients with diabetes mellitus commenced on antidepressants experienced similar changes in BMI as patients who did not have diabetes.

## Method

We used an EMIS Web search (www.emishealth.com/products/mental-health) within our general practice in Larkhall and Stonehouse, Scotland, UK, to identify patients on the antidepressants of interest. The practice population comprised 11 994 patients, of whom 232 patients were on mirtazapine, 456 on citalopram, 353 on fluoxetine and 221 on sertraline. We excluded those who did not have recorded start and current BMIs. Body mass index for each antidepressant was expressed as a percentage increase upon the start BMI. The duration of treatment and any prior antidepressants used were recorded to discern whether the antidepressant of interest had been prescribed for a relatively short period of time and whether any previous antidepressant may have influenced the changes. A diagnosis of diabetes mellitus made by the general practice was also noted. The project was registered with NHS Lanarkshire's Clinical Quality Department. The results were tabulated using Excel 2007 for Microsoft Windows, which was also used for the majority of statistical analysis. For nominal data, one-way analysis of variance (ANOVA) and corresponding two-tailed *t*-tests were used. For categorical data, the χ^2^ statistic was used. Spearman's rho correlations were used to examine the relationship between BMI change and duration of treatment.

## Results

**Fig. 1:**
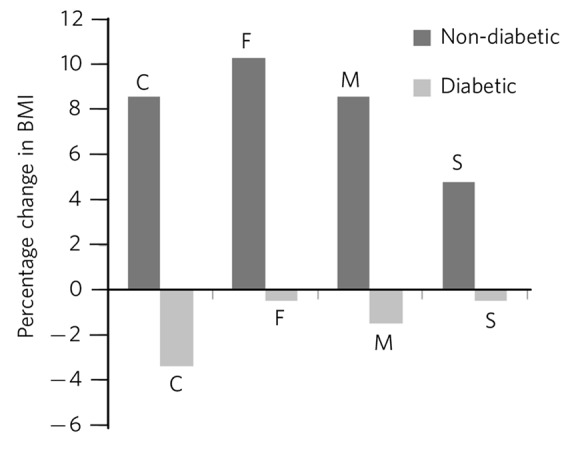
Percentage change in BMI, comparison of patients with diabetes and patients with no diabetes. C, citalopram; F, fluoxetine; M, mirtazapine; S, sertraline; F_(7,260)_ = 34.7, *P*<0.001.

Table 1 shows demographic and clinical measures for each antidepressant group. More females were prescribed the four antidepressants of interest, however, the male/female ratio did not vary significantly between antidepressants (χ^2^ = 4.1, d.f. = 3, *P* = 0.25). Patients prescribed fluoxetine and citalopram were younger than those prescribed mirtazapine and sertraline (F_(3,264)_ = 17.1, *P*<0.001). The duration of treatment was longest with fluoxetine (F_(3,264)_ = 19.0, *P*<0.001). Around two-thirds of all patients showed an overall BMI increase associated with treatment, with no differences between antidepressants (χ^2^ = 1.4, d.f. = 3, *P* = 0.7). The BMI at the start of treatment did not differ significantly between antidepressants for patients with (F_(3,207)_ = 0.13, *P* = 0.9) and without diabetes (F_(3,58)_ = 1.04, *P* = 0.38). Mean increases in BMI were seen for patients who did not have diabetes and mean decreases were seen for patients who had diabetes (Fig. 1). No statistical differences for the increases or decreases were noted between antidepressants, although percentage BMI gain from baseline was greatest for fluoxetine (10.3%) for patients without diabetes. At the commencement of treatment the ratio of normal weight (BMI <25 kg/m^2^) to abnormal weight (all other BMI categories) was 1:2 for patients who did not have diabetes and 1:8 for patients with diabetes (χ^2^ = 13.0, d.f. = 1, *P*<0.001). Looking at antidepressant history (Table 2), patients prescribed mirtazapine were more likely to have been treated with another antidepressant beforehand (χ^2^ = 11.6, d.f. = 3, *P*<0.01).

**Table 1:** Demographic and clinical measures for patients treated with antidepressants

	Citalopram (*n* = 68)	Fluoxetine (*n* = 66)	Mirtazapine (*n* = 66)	Sertraline (*n* = 68)	*P*	*F*
*All patients*						
Male, *n* (%)	20 (29)	20 (30)	29 (44)	26 (38)	0.25	
Age at start of treatment, years: mean (95% CI)	50.8 (47.8–53.7)	46.8 (43.5–50.1)	57.5 (54.2–60.8)	63.1 (59.1–67.2)	< 0.001	*F*_(3,264)_ = 17.1
Duration of treatment, years: mean (95% CI)	7.0 (6.0–7.9)	8.8 (7.5–10.2)	5.1 (4.2–6.1)	3.6 (2.9–4.3)	< 0.001	F_(3,264)_ = 19.0
BMI increase	42 (62%)	47 (71%)	43 (65%)	46 (68%)	0.70	

*Patients without diabetes, n* (%)	56 (82)	51 (77)	50 (76)	56 (82)		
BMI start, kg/m^2^: mean (95% CI)	28.9 (26.9–30.9)	28.4 (26.6–30.1)	28.1 (26.5–29.7)	28.9 (26.9–30.9)	0.9	F_(3,207)_ = 0.13
BMI range, *n* (%)	15–53	18–54	14–42	15–53		
<25	23 (41)	18 (35)	13 (26)	23 (40)		
25–30 (overweight)	14 (25)	17 (33)	20 (40)	15 (27)		
30–40 (obese)	15 (27)	15 (29)	16 (32)	14 (25)		
40+ (very obese)	4 (7)	1 (2)	1 (2)	4 (7)		
BMI change, mean (95% CI) Kg/m^2^	2.3 (1.2–3.4)	2.8 (1.7–4.0)	2.2 (1.2–3.2)	1.5 (0.7–2.4)	0.24	F_(3,197)_ = 1.43
Percentage	8.6 (4.8–12.3)	10.3 (6.1–14.4)	8.6 (4.9–12.3)	4.8 (1.9–7.7)	0.25	F_(3,196)_ = 1.38

*Patients with diabetes mellitus,* *n* (%)	12 (18)	15 (23)	16 (24)	12 (18)		
BMI start, kg/m^2^: mean (95% CI)	29.0 (26.6–31.3)	32.5 (29.2–35.8)	31.5 (29.0–34.1)	28.0 (26.6–31.3)	0.38	F_(3,58)_ = 1.04
BMI range, *n* (%)	21–35	24–52	18–40	21–35		
< 25	2 (17)	1 (7)	1 (6)	2 (17)		
25–30 (overweight)	5 (42)	4 (27)	4 (25)	5 (42)		
30–40 (obese)	5 (42)	9 (60)	10 (63)	5 (42)		
40+ (very obese)	0 (0)	1 (7)	1 (6)	0 (0)		
BMI change, mean (95% CI)						
Kg/m^2^	−1.1 (−2.7 to 0.5)	−0.4 (−2.3 to 1.6)	−0.69	−0.6 (−2.2 to 1.0)	0.95	F_(3,50)_ = 0.11
Percentage	−3.4 (−9.0 to 2.3)	−0.5 (−6.8 to 5.8)	(−1.99 to 0.61) −1.5 (−5.7 to 2.6)	−0.5 (−5.7 to −4.8)	0.88h	F_(3,48)_ = 0.21
Blood glucose, mmol/L: mean (95% CI)						
Start	11.0 (7.8–14.1)	7.4 (5.5–9.2)	13.1 (10.1–16.1)	10.2 (6.6–13.9)		
Current/last	11.7 (7.8–15.6)^a^	12.0 (7.1–17.0)^a^	13.1 (9.1–17.2)^b^	11.7 (8.3– 15.1)^c^		

BMI, body mass index.

a. *n* = 6.

b. *n* = 12.

c. *n* = 9.

**Table 2:** Antidepressant history

	Current antidepressant			
Previous antidepressant	Citalopram (*n* = 68)	Fluoxetine (*n* = 66)	Mirtazapine (*n* = 66)	Sertraline (*n* = 68)
None, *n* (%)	33 (49)	30 (45)	16 (24)	21 (31)

Citalopram, *n* (%)	–	16 (24)	23 (35)	18 (26)
Treatment duration, years (range)	–	0.7 (0.08–4.0)	1.0 (0.08–12.0)	3.0 (0.16–12.0)

Fluoxetine, *n* (%)	12 (18)	–	8 (12)	15 (22)
Treatment duration, years (range)	0.5 (0.08–10.0)	–	2.0 (0.4–5.0)	0.5 (0.16–13.0)

Mirtazapine, *n* (%)	7 (10)	7 (11)	–	4(6)
Treatment duration, years (range)	0.25 (0.08–1.0)	1.5 (0.08–5.0)	–	1.5 (0.08–4.0)

Sertraline, *n* (%)	2 (3)	1 (2)	2 (3)	–
Treatment duration, years (range)	0.7 (0.16–1.2)	0.08	0.16 (0.08–0.25)	–

Venlafaxine, *n* (%)	3 (4)	4 (6)	7 (11)	3 (4)
Treatment duration, years (range)	0.25 (0.16–7.0)	1.0 (0.08–3.0)	2.4 (1.0–13.1)	1.0 (0.5–1.0)

Other, *n* (%)	11 (16)	7 (11)	10 (15)	7 (10)
Treatment duration, years (range)	2.3 (0.16–7.0)	2.0 (0.08–7.0)	2.6 (0.16–9.1)	1.0 (0.08–7.0)

**Fig. 2:**
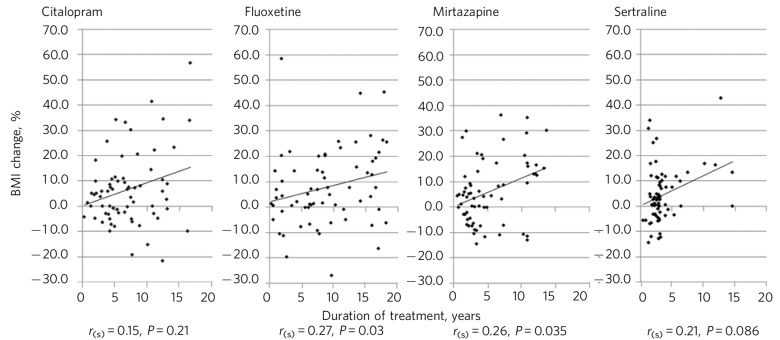
Percentage BMI change and duration of treatment with Spearman's rho correlations. *r*_(s)_, Spearman's rho.

Figure 2 shows Spearman's rho correlations for percentage BMI change with time. Significant positive correlations were seen with fluoxetine (*P* = 0.03) and mirtazapine (*P* = 0.04). For mirtazapine and sertraline BMI reductions were associated with short duration of treatment.

## Discussion

Long-term antidepressant treatment with citalopram, fluoxetine, mirtazapine and sertraline was associated with an increased BMI in approximately two-thirds of patients. There were differences between patients with and without diabetes. In patients with diabetes, we noted a mean reduction in BMI with all four antidepressants, whereas in patients without diabetes there was a mean increase in BMI. Notably, a long duration of antidepressant treatment was seen, in some patients extending to almost two decades. BMI increases were positively correlated with duration of treatment, reaching statistical significance for fluoxetine and mirtazapine. Our primary hypothesis was not supported, in that mirtazapine was not associated with the greatest increase in BMI compared with the other three antidepressant medications.

### Comparison with existing literature

Diabetes was relatively common among patients in each of the antidepressant groups, at 18–24%, which was significantly higher than the national average of 6% (www.diabetes.co.uk/diabetes-prevalence.html). This may have been related to a number of factors, including the increased likelihood of depression in patients with physical health comorbidity. In addition, patients with diabetes are usually offered regular reviews for their illness and may have had more opportunity to discuss mental health problems such as depression. Some studies have suggested that antidepressant prescription is associated with an increased relative risk of type 2 diabetes, although the elevation in absolute risk was modest.^3^ From our data, patients with diabetes were more likely to be overweight at the commencement of treatment and also as a group to lose a small proportion of weight following treatment. The mechanism for this may be that by treating depression, motivation, ability to exercise and general physical health improved. Another possibility is that people with diabetes were more likely to attend regular review/annual health checks, therefore risk factors such as obesity were more proactively managed. There was a suggestion that fluoxetine was given to diabetic patients with the highest BMI – perhaps the result of channelling bias,^9^ where prescribers actively, or perhaps subconsciously, selected patients who had a relatively high BMI to start with as being suitable for treatment with fluoxetine.

Weight change is a common clinical feature of depression and at first glance increasing weight associated with treatment may, for some, seem clinically favourable. However, our patients had a mean BMI of 30.1 kg/m^2^ before the start of their antidepressant medication, 2.9 kg/m^2^ higher than the Scottish average for adults, which was 27.2 kg/m^2^ in 2012.^10^ Our total population was by definition overweight,^11^ that is, their BMI was 25 kg/m^2^ or above, even prior to treatment. Review of the existing literature suggested that mirtazapine was likely to cause more weight gain than other antidepressants,^1^ but our data did not fully support this position – surprisingly, we found that fluoxetine caused the greatest proportional increase in BMI. However, fluoxetine was associated with a longer duration of treatment, which may confound this finding. This position with fluoxetine is of interest given its historical use in the treatment of obesity^12^ and bulimia nervosa (see the *British National Formulary*).

Interestingly, patients prescribed mirtazapine were more likely to have been treated with another antidepressant beforehand. This may be related to prescriber caution regarding adverse effects with mirtazapine, such as sedation and weight gain. In addition, mirtazapine was the most recently available antidepressant of the four in question.

### Strengths and limitations

There were some important limitations to this study. This was a retrospective observational study, therefore we cannot assume causality. We excluded patient records which did not have two sets of BMI recordings within the past 3 years. We may therefore have introduced selection bias by excluding those who did not attend for regular review. For the same reason, we may have targeted more patients with diabetes as there was a requirement, drawn from the Quality Outcome Framework (QOF),^13^ for this population to have at least annual reviews, hence they would be included in our dataset of the past 3 years. There were additional confounding factors such as comorbidity, previous antidepressants, and other prescriptions such as antipsychotics, steroids and thyroid medications, which may have had an effect on weight gain. It is also possible that patients may put on weight as part of the normal process, which is a further potentially confounding factor. There were only a small number of patients who had two separate blood glucose measurements taken, therefore we suggest that blood glucose results are interpreted with caution. Having mentioned limitations, our data do reflect a clinically relevant population sample and add utility in informing prescribers and patients about specific metabolic effects associated with antidepressants.

### Implications for clinical practice

Antidepressant prescription is common in general practice. Data from Information Services Division (ISD) Scotland^14^ showed that antidepressant prescribing continued to rise between 2009/2010 and 2010/2011. The rate of growth increased from 7.6% in 2009/2010 to 8.1% in 2010/2011. Daily use of antidepressants has grown from 6.9% in 2001/2002 to 11.3% of the population (aged 15+) by 2010/2011. We considered the long-term treatment pattern for specific antidepressants and its potential risks. We suggest formal clinical guidelines for continuing treatment beyond the period of, for example, 2 years and consider including such long-term treated patients in the general practice register of those with serious mental illness. It is known that weight change effects for fluoxetine are weight loss in the acute phase (less than 1-year period) and weight gain thereafter.^2^ Prescribers should be more mindful of the metabolic impact of all antidepressant medications, particularly when long-term treatment is concerned, and perform regular recordings of weight and blood glucose on all long-term patients with mental disorders, not just those with diabetes mellitus. It is likely that social, environmental and illness factors, as well as medications, play a significant role in weight changes. Perhaps encouraging healthy choices with all patients rather than targeting dietary and healthy living advice at those taking medications traditionally associated with weight gain is a more rounded approach. The finding that patients with diabetes show decreases in BMI requires further investigation and replication with a larger sample size and other antidepressants.
